# Fowne’s Manual of Chemistry

**Published:** 1878-08

**Authors:** 


Fownes' Manual of Chemistry, theoretical and practical, revised and corrected by Henry Watts, B.A., F.R.C. A new American from the twelfth English edition. Edited by Robert Bridges, M.D., Professor of Chemistry in the Philadelphia College of Pharmacy. With one hundred and seventy-seven illustrations. Philadelphia: Henry C. Lea. 1878.

This deservedly popular chemistry having undergone revision and correction to suit the progress of science, will most certainly find favor with teachers as a text-book for students.

